# US regulations to curb alleged cancer causes are ineffectual and compromised by scientific, constitutional and ethical violations

**DOI:** 10.1007/s00204-022-03429-5

**Published:** 2023-04-08

**Authors:** Gio B. Gori, Michael Aschner, Christopher J. Borgert, Samuel M. Cohen, Daniel R. Dietrich, Corrado L. Galli, Helmut Greim, John S. Heslop-Harrison, Sam Kacew, Norbert E. Kaminski, James E. Klaunig, Hans W.J. Marquardt, Olavi Pelkonen, Ruth Roberts, Kai M. Savolainen, Aristidis Tsatsakis, Hiroshi Yamazaki

**Affiliations:** 1 Emeritus Director, The Health Policy Center, Bethesda, Maryland USA; 2 Formerly Deputy Director, Division of Cancer Cause and Prevention, National Cancer Institute, Bethesda, Maryland USA; 3 Professor of Molecular Pharmacology, Albert Einstein College of Medicine, Bronx, NY 10461 USA; 4 Applied Pharmacology and Toxicology, Inc., Department of Physiological Sciences, University of Florida College of Veterinary Medicine, Gainesville, FL, USA; 5 Havlik Wall Professor of Oncology, Department of Pathology and Microbiology and Buffett Cancer Center, University of Nebraska Medical Center, Omaha, NE 68198-3135 USA; 6 Professor of Human and Environmental Toxicology, Dean of Studies, Faculty of Biology, Konstanz University, Konstanz, Germany; 7 Professor of Toxicology and Risk Assessment, Department of Pharmacological and Biomolecular Sciences, University of Milan, 20133 Milan, Italy; 8 Professor emeritus of Toxicology and Environmental Health Technical, University of Munich, Munich, Germany; 9 Department of Genetics and Genome Biology, University of Leicester, Leicester, UK; 10 McLauglin Centre for Population Health Risk Assessment, University of Ottawa, Ottawa, Ontario K1N6N5 Canada; 11 Pharmacology & Toxicology, Director, Institute for Integrative Toxicology, Michigan State University, East Lansing, Michigan USA; 12 Professor of Environmental Health, Indiana University, Bloomington, Indiana 47408 USA; 13 Department of Experimental & Clinical Toxicology, University Hamburg Medical School (Retired), Hamburg, Germany; 14 Professor of Pharmacology (Retired), Research Unit of Biomedical Sciences/Pharmacology and Toxicology, University of Oulu, Oulu, Finland; 15 Apconix Ltd. Chair and Director of Drug Discovery, University of Birmingham, Birmingham, UK; 16 Finnish Institute of Occupational Health, Helsinki, Finland; 17 Chairman of Toxicology and Forensics Departments, University of Crete Medical School, Heraklion, Crete Greece; 18 Drug Metabolism and Pharmacokinetics, Showa Pharmaceutical University, Machida, Tokyo 194-8543 Japan

## Abstract

The 1958 Delaney amendment to the Federal Food Drug and Cosmetics Act prohibited food additives causing cancer in animals by appropriate tests. Regulators responded by adopting chronic lifetime cancer tests in rodents, soon challenged as inappropriate, for they led to very inconsistent results depending on the subjective choice of animals, test design and conduct, and interpretive assumptions. Presently, decades of discussions and trials have come to conclude it is impossible to translate chronic animal data into verifiable prospects of cancer hazards and risks in humans. Such conclusion poses an existential crisis for official agencies in the US and abroad, which for some 65 years have used animal tests to justify massive regulations of alleged human cancer hazards, with aggregated costs of $trillions and without provable evidence of public health advantages. This article addresses suitable remedies for the US and potentially worldwide, by critically exploring the practices of regulatory agencies vis-á-vis essential criteria for validating scientific evidence. According to this analysis, regulations of alleged cancer hazards and risks have been and continue to be structured around arbitrary default assumptions at odds with basic scientific and legal tests of reliable evidence. Such practices raise a manifold ethical predicament for being incompatible with basic premises of the US Constitution, and with the ensuing public expectations of testable truth and transparency from government agencies. Potential remedies in the US include amendments to the US Administrative Procedures Act, preferably requiring agencies to justify regulations compliant with the Daubert opinion of the Daubert ruling of the US Supreme Court, which codifies the criteria defining reliable scientific evidence**.** International reverberations are bound to follow what remedial actions may be taken in the US, the origin of current world regulatory procedures to control alleged cancer causing agents.

**ENDORSERS of this paper**

**Hermann M. Bolt**^1^, **Wolfgang Dekant**^2^, **José Luis Domingo**^3^, **Jay I. Goodman**^4^, **Angelo Moretto**^5^, **Emanuela Testai**^6^, **Nico P.E. Vermeulen**^7^, **Kendall B. Wallace**^8^

## Introduction

“We the People of the United States” constituted the US government to serve their quest for freedom and the pursuit of happiness.^1^ In contrast to the millennial tradition of authoritarian government models prevailing in the world today, the historical innovation of the United States is the assignment of national sovereignty to the people and not to government operatives. The clear implication for the ethical behavior of public servants in government is to be transparent and truthful, for citizens shall not be deceived.

Still, motivated by natural instincts to prevail, official regulatory agencies have continually labored to achieve autonomous authority, obtained in the US by statutory authorization to emit regulations with the power of law, and in most other countries by deferential legislative approval of agency protocols. On these grounds, regulatory agencies in the US and around the world set out to police the ambitions of special interests and to modify choices and behaviors of free citizens.

Such broad agendas include health, safety and environmental regulations to prevent cancer risks in humans. Such regulations require evaluations of potential human cancer hazards from natural and artificial molecules ubiquitous in the environment: a set of substances which delimits the scope of the current paper. At prevailing low exposures, these agents offer no discernible evidence of adverse effects regarding cancer risk. Overall, regulations would not be warranted if such natural low exposures are not exceeded, but could become of interest when duration and intensity of exposure may exceed thresholds of no-observable adverse effects. Hence, the science of toxicology and scores of professionals eager to sustain or bar policies and regulations, laboring for transparent, truthful, testable, and measurable scientific evidence about hazards and thresholds of interest.^2^

## Standards of reliable evidence

What are the standards of evidence appropriate for official regulation? During the last decades, much has been written recommending the use of evidence in public life. The US National Library of Medicine carries over a half million publications on evidence-based medicine, toxicology, epidemiology and a host of other disciplines. No extant paper, however, has attempted to highlight the essential criteria defining what is truthful, reliable, and thus functional scientific evidence, ready for effective technologies and sensible public and personal policies.

Here, toxicology being an experimental natural science, truthful evidence needs qualification. Unlike the absolutes of purely cerebral disciplines such as mathematics, geometry or formal logic, all experimental natural sciences postulate empirical truths in a probabilistic context, due to the inherent approximations of observations and measurements, the multitude of potentially confounding variables and the natural vagaries of atoms, molecules, radiations and overall matter.

Although nominally provisional, such truths—or natural laws of experimental science—are confirmed by the operational success of countless technological applications: wholesome foods and safe water are the norm, innumerable chemicals are safely used, personal and occupational hygiene keep us healthy, medicines and vaccines cure, and so on. Undeniably, the factual reliability of technological applications vouches for their foundation on true scientific evidence: a context where toxicology is also expected to warrant true scientific evidence in justifying public policies and regulations. Such evidence, as common to all natural sciences, is developed, tested and approved in two separate phases:

- A research activity dealing with knowledge-in-the-making and dedicated to test hypotheses and theories about the mechanics of the physical world.
- A body of tested and self-standing knowledge ready for technology and policy applications, and to inspire new testable hypotheses and theories.

Research hypotheses are considered scientific in the context of prospecting for and motivating scientific research, but are not part of validated scientific knowledge. Venture capital may support testing of research hypotheses hoping for the big win with no guarantee of success, but no sensible entrepreneur planning to market functional applications would rely on hypotheses of any kind. Similarly, it should be unethical and forbidden for official agencies to consider hypotheses of alleged hazards in support of regulations, especially when such would massively hamper national economies, influence the anxieties, choices and behavior of billions of people, entail heavy penalties and even detention for hapless transgressors. Only hazards and risks certified as materially exceeding no effect thresholds, according to verified experimental evidence, could justify compulsory government regulation.^3^^,^ 4

In obtaining such verified evidence, toxicology would be expected to follow the scientific method. Much has been written about the method’s philosophical underpinnings, but the method and science would be powerless without operational standards to secure reliable measurements and empirical controls: the crucial starting points toward factual knowledge. Aware of this essential need, experimental scientists would recognize the following evidentiary warrants, which must be met as minimum requirements before hypotheses may aspire to become reliable operational knowledge:

- What is measured is relevant to the hypotheses being tested.
- Measurements are authentic: what is measured is what is declared to have been measured.
- Measurements have measured and quantified error rates small enough to ensure observational and statistical consistency.
- Experimental methods, procedures and tools are relevant and peer-trusted in testing the hypotheses being considered.
- Known externalities capable of confounding measurements and results are quantified and controlled.
- Sufficient ground controls allow counterfactual inferences.
- The experimental record and conclusions are published in peer-reviewed journals.
- Detailed procedural descriptions and original data are disclosed.
- Results are reproducible and supported by the above lines of evidence.

These standards are not abstruse philosophical propositions, but sensible logical yardsticks implicitly or explicitly observed in common human transactions. Pumping gasoline at a station implies confidence about sequential gallon measurements differing imperceptibly, fuel being genuine and not adulterated, fuel grade being as selected, the price posted being correct, the effects of externalities—such as temperature—being negligible, that such conditions apply to all customers, and so on.

Moving to a scientific scenario, precisely assessing the temperature causing water to boil requires first ascertaining the accuracy of the instruments employed, toward being confident of negligible error rates of measurement. Next, experimental controls would require checking the possible influence ambient temperatures and barometric pressure; controlling and specifying the heat source and location, the shape and composition of the vessel in relation to heat sources and other environmental exposures. The purity of the water needs to be ascertained to control the interference of extraneous molecules dissolved. Further observations may include the location of instruments, the amount of water relative to the amount of heat applied, how heat is applied, and several other details.

As transparent proofs of honesty, the warrants described are clearly self-evident and the essence of operationally spotless scientific research. Short of them, experiments and observations are stuck as untested conjectures, not to be trusted in enabling functional technologies nor fair policies and regulations. In fact, it is these operational standards of experimental measurement and control which allowed science to accumulate a body of empirical knowledge sufficiently certain to enable the countless technologies currently gracing human lives.

These standards have been codified in legal detail by the 1993 Daubert verdict of the US Supreme Court, which defines what scientific evidence is reliable and admissible in federal courts.^5^ Written in the high legalese of Supreme Court reports, the original Daubert decision is no easy reading for the unschooled, but several summaries highlight the core points of the ruling. Essentially, Daubert requires admissible testimonial evidence to be certified as follows:

- The primary experimental and observational data have negligible error rates objectively measurable and explicitly measured.
- The experimental and observational data supporting the evidence presented are relevant to the issues at hand.
- The evidence presented has been tested by experimental and observational methods generally accepted by the scientific community.
- The evidence presented has been subjected to peer-review and publication.

While compiled for lawyer’s eyes, these criteria accord with the standards of scientific evidence previously listed, and together define what evidence is applicable to sustain defensible policies and regulations.

Such standards, however, have not been and are not officially adopted to justify the most costly health, safety and environmental regulations, which instead are imposed by indifferent authority disguised as scientific, but in fact driven by default assumptions contrary to science and objective evidence. As a telling instance, what follows focuses on the official setup of experimental animal tests presumed to identify human cancer hazards, and widely used to justify regulatory activities in the US and around the world.

## General considerations on testing alleged chemical cancer hazards

Human testing of presumably hazardous chemical cancer hazards is considered unethical, such testing being mostly run in rats and mice.^6^ A distinction needs to be made of short-term and long-term animal tests, the latter designated as lifelong chronic bioassays or simply bioassays. Short-term or acute animal tests—lasting less than 90 days—offer verifiable insights on short-term effects in animals and humans. Assisted by pharmacokinetic data, such tests can reasonably predict the near term absorption, internal distribution, metabolism and excretion rates of natural or synthetic agents in animals and humans. They can also determine acute no effect thresholds: namely, the physiologic concentrations and exposure conditions below which short-term adverse effects are not observed. Such evidence allows the effective regulation of safe levels of short-term exposure in animals and humans under different scenarios. This is feasible, because adverse effects occurring in less than 90 days generally concern basic and stable physiologic mechanisms conserved in many species including humans; mechanisms not significantly modified over short time by the multiple and random confounders affecting long-term bioassays.

In fact, such random confounders and other considerations deny the credibility of lifetime animal bioassays as human surrogates for conditions associated with chronic exposures, such as cancer, cardiovascular and neurological diseases, immunity and endocrine disorders and more. Protracted effects in such bioassays evolve randomly over lifetimes and through multiple, incidental, unpredictable, hypothetical and usually unknowable modes of action. Somatic and behavioral conditions, life history, disease sensitivities, hormonal differentials; genetic, epigenetic and immune repair efficiencies; environmental and dietary adaptations and other disparities in different animals, all and more diverge into different causal opportunities ostensibly unique to individual cancer pathologies. Indeed, the outcomes of chronic bioassays are inconsistent across animal species, among strains of the same species, and even among individuals of the same inbred strain.

Ultimately, the core problem is the absence of valid methods for an objective translation of chronic animal data into verifiable forecasts of human hazards and risks. Yet, ignoring this blunt reality, lifetime cancer bioassays for the last 65 years have been and are still officially prescribed in the US and worldwide. They support unprecedented regulations imposing $trillion costs to world economies, misguided political and social policies and the unfathomable costs of unwarranted public anxieties, while incapable of showing testable proofs of their public health or environmental utility.

## The animal lifetime bioassay for human cancer hazards

The archetypal lifetime bioassay for carcinogens is a standardized test in rats and mice, requiring over 3 years at a cost of several $million per test. Its use follows the 1958 Delaney Clause of the federal Food, Drug, and Cosmetics Act, prohibiting food additives if they cause cancer in animals by appropriate tests. The statute is silent about what makes appropriate tests, and regulators responded by unilaterally adopting default bioassays in rodents. They were soon challenged, because—as just described—bioassays yield widely contrasting results depending on animal species and inbred strain choices, design, setup, conduct and interpretation rules. Decades of discussions and trials have finally come to accept the overwhelming evidence excluding the feasibility of chronic animal tests to factually infer cancer hazards and risks in humans.^7^ Even so, the same bioassays continue as the mainstay of oppressive regulations.

The intent of the Delaney clause was rapidly extended beyond foods without statutory authorization, to regulate every conceivable exposure in lifestyles, housing, agriculture, medicine, communications, transportation, industry, defense and more. More poignant still, such practices are not exclusive to the US but also migrated to the world, sharing a common philosophy, assumptions and terminology and becoming a regulatory praxis of the World Health Organization.

To perceive hands-on the roots of those massive interventions, let it be repeated how cancer bioassays yield different results depending on their set up and conduct, what animal’s numbers and susceptibilities, what exposures and doses, diets, environmental conditions, diseases and more. In fact, shortly after the Delaney clause became law, the US Food and Drug Administration (USFDA) found traditional staple foods to be causing cancer in animal tests. Prohibiting basic constituents of human diets would have been unthinkable, and the agency was forced to secure from Congress a dispensation for a special category of products defined as Generally Recognized as Safe (GRAS). Such clear warning about the inevitably arbitrary makeup of any chronic animal test was ignored when other agencies—notably the US Environmental Protection Agency (USEPA)—embarked into massive regulatory programs in the early 1970s, unilaterally seizing the Delaney mandate as their principal instrument.

As the core example for other agencies, the USEPA guidelines to initiate and run lifetime bioassays in rats and mice have been issued without legislative concurrence, imposing unilaterally a standard set of arbitrary default assumptions. The agency openly admits such assumptions are designed to facilitate its regulatory agenda by maximizing positive cancer responses in animals, offering the oblique justification of acting under a mandate to regulate by proactive precaution.^8^ Such mandate, however, is absent in US law, for it would grant authority of imposing unlimited economic burdens on the commonwealth based on alleged grounds.^9^ In practice, the agency uses default assumptions to write and enforce its own rules of evidence, leading to the contrived bioassay for the classification of putative cancer hazards, in turn regulated under the pretense of precaution.

The most significant default assumptions are as follows: inbred rats and mice must be accepted as equivalent proxies of freewheeling humans; bioassay must be run for the lifetime of the animals at maximum tolerated doses (MTD), which are expected to generate a chronic general toxicity measurable as a lifetime 10% decreased body weight gain; bioassay interpretation must assume that excessive lifetime MTD animal exposures represent humans exposed to much smaller doses and at fractional durations; the metabolism in animals at MTD is assumed equivalent to the metabolism of humans at low doses and exposure duration; benign lesions are assumed equivalent to malignant ones; the route of exposure is assumed to be irrelevant; linear extrapolations from high MTD doses in animals to lower doses in humans must be used. The statement “Animal studies are conducted at high doses in order to provide statistical power” disguises the agency resolve to artificially maximize cancer instances under MTD conditions. The clear intent is to produce cancer signals by whatever means, aiming at precautionary regulations.

Imposed with the force of law, such assumptions have reduced chronic regulatory bioassays into a formulaic paradigm inhibiting introspection, while openly admitting the absence of objective, truthful and relevant evidence in the entire process. The International Agency for Research on Cancer, an arm of the World Health Organization, conceded 50 years ago *“…a correlation between carcinogenicity in animals and possible human risk cannot be made on a scientific basis.*” In 1981, Dr. David Rall, first director of the National Toxicology Program, testified in congress that human cancer risks could not be derived scientifically from animal tests, insisting that Congress and the public ought to have faith (*sic*) in experts who comb rat and mouse entrails to divine human cancer hazards. A 2009 committee report of the US National Academy of Sciences reads “… *the defaults involving science and policy judgments, such as the relevance of a rodent cancer finding in predicting low-dose-human risk, are used to draw inferences ‘beyond the data’, that is beyond what may be directly observable through a scientific study.*”^10^

In the same document (p.17), the Academy’s committee concluded “*... risk management decisions continue to be made by state and federal agencies; however it is not known whether the decisions being made are health protective.*” While those decisions could also be damaging to public health, the Academy’s committee and regulatory agencies appear fully aware that science and commonsense preclude lifetime bioassays in animals to be predictive of human cancer hazards and risks.

Be as it may, bioassays are only a first step in the current process of regulating alleged cancer risks. The second move is the mathematical extrapolation from lifetime MTD data in rodents to the lower natural exposures in humans, raising the question of what mathematical model should be appropriate for the task. Decades ago, US regulators fitted lifetime animal MTD data to various mathematical models such as the Benchmark dose, One hit, LMS, constrained LMS, maximum-likelihood LMS, Weibull, constrained Weibull, Logistic, Probit, Poisson and more models and variants. Each model represents a different x/y distribution and a specific plot or graphic visualization of its mathematical function.

Impasses soon emerged, for there are no logical or mechanistic grounds to prefer one model over others, while the dose data of any MTD bioassay could be made to fit most any model. Different models, however, lead to low-dose extrapolations differing by several orders of magnitude. Facing a choice, the USEPA imposed the linear model as the default assumption to be used, not because of any objective rhyme or reason but because it seemed the closest to suit agency policy, while disguising arbitrariness with mathematical sleight.

Pained to appear dictatorial, regulatory agencies in the US and around the world also have sought to achieve an aura of consensus by submitting bioassay and modeling records to advisory committees. By appointing such committees as advisory but without executive clout, regulators have aptly managed dissenting opinions internally while freely pursuing their interest in proliferating alleged human cancer hazards.

A final injection of bias comes with the quantitative interpolation of safety factors during rulemaking. Naturally arbitrary, such factors contribute a major obfuscation, as they escape scrutiny under the seduction of precaution and the hollow authority of precedent. They are numerical adjustments interpolated during the final editing of a rule according to political checks and balances, and toward ruling for the minimal exposures compatible with projected uses. Obviously, such minimal exposures could be defined without default bioassays, magic dose–response models, advisory committees and safety factors. Defined by a balanced analysis of factual commonwealth benefits, minimal precautionary exposures could result from an accounting process pragmatic and transparent enough to be an effective discipline to market ambitions and a safeguard for public health.

In summary, currently enforced chronic bioassays to define human cancer hazards and risks do not meet the evidentiary standards of experimental science or the Daubert standards of admissible evidence. They are run in irrelevant animal models, according to irrelevant and intentionally forced experimental designs and default assumptions, do not control for externalities, and their results are manipulated by arbitrary mechanistic models, perfunctory advisory committees and safety factors.^11^ The process openly trashes the requirements of objective evidence as it trashes commonsense, while claiming an inexistent mandate of precaution to cover for a mandate of accountability. At the core, the process flouts the US Constitution’s injunction that citizens shall not be deceived.

In a curious turn of events, the alleged cancer hazards produced by the USEPA have been embraced as their flags by assorted advocacy groups, making the agency a super-advocacy organization with the awesome power of regulating with the force of law.

As noted, with few rhetorical modifications, these very practices found acceptance with regulators worldwide, after earlier adoptions by the International Agency of Research on Cancer (IARC), an agency of the World Health Organization (WHO), and by the major economic blocks of the European Union, Japan, China and South America. Most scientists have embraced such practices without protesting, even though lesser transgressions have led to harsh punishments for misconduct in their ranks. More remarkable is how societies pretending to be free, rational and enlightened, have been pressed to countenance and fund such openly misleading, costly and fruitless practices at the highest levels of public policy. Even more remarkable is how legislators of free democratic nations could overlook obvious conflict of interest ambiguities in statutes setting up and motivating regulatory agencies, thus enticing regulators into deceptive gambits.

## US government stance

The natural aspiration of US government staff has been to operate autonomously under the traditional and autocratic Crown-endorsed authority of most European bureaucracies: a model the US constitution disallows. Such aims arose repeated concerns, more recently during the administration of Franklin D. Roosevelt, when numerous executive agencies with independent authorities were set up to sustain New Deal policies, and more after Roosevelt vetoed the 1940 Walter-Logan bill calling for court supervision of those agencies. The onset of WW2 delayed further discourse, soon revived with the passage of the 1946 Administrative Procedure Act (APA).^12^

In principle, the APA details the constitutional behavior expected from government agencies, but a close examination shows key agencies endowed with independent legislative, enforcement and judicial authority, further protected by the deference of state and federal courts. Feeling sovereign, the agencies’ behavior is more in line with those “*ancien régime*” practices the US Constitution excludes. The APA briefly disposes of the central issue of what evidence can justify the agencies independent promulgation of legally binding regulations, offering this equivocal directive:

“*Except as otherwise provided by statute, the proponent of a rule or order has the burden of proof. Any oral or documentary evidence may be received, but the agency as a matter of policy shall provide for the exclusion of irrelevant, immaterial, or unduly repetitious evidence. A sanction may not be imposed or rule or order issued except on consideration of the whole record or those parts thereof cited by a party and supported by and in accordance with the reliable, probative, and substantial evidence*.”^13^

Contrary to constitutional writ, the Act admits the autonomous authority of agencies to issue rules and orders with the executive force of laws promulgated by Congress, burying unresolved Constitutional issues. Further, the Act identifies proponents of rules and orders from outside of agencies, but does not touch on the more frequent reality of rules and orders originating within the agencies and teeming with conflicts of interest. It also fails to provide guidance on what may authorize the exclusion of “*irrelevant, immaterial, or unduly repetitious evidence*”, nor of what may constitute “*reliable, probative, and substantial evidence*”. In essence, agencies are given carte blanche in gathering whatever evidence suits their policy, as it happened with the imposition of default assumptions, the choice of extrapolating mathematical models, the selectively appointed review committees, and the insertion of safety factors capping the abusive “regulatory science” of alleged human cancer hazards.

Challenging agency rules is notoriously difficult, for US agencies also have their own administrative courts first in line to adjudicate external grievances, have their own prosecutors and judges with the power to issue fines and detention, and a fully armed police ready for action. An external party failing to obtain relief in administrative trials can challenge agencies in federal courts, but success has been extremely rare. Still, it is encouraging to find the APA listing those agency misbehaviors a court would find unlawful enough to:

“*... set aside agency action, findings, and conclusions found to be (1) arbitrary, capricious, an abuse of discretion, or otherwise not in accordance with law; (2) contrary to constitutional right, power, privilege, or immunity; (3) in excess of statutory jurisdiction, authority, or limitations, or short of statutory right; (4) without observance of procedure required by law;* *(5) unsupported by substantial evidence....; or (6) unwarranted by the facts...* ″.^14^

On these counts, US agencies’ actions should have been found illegal long ago in the regulation of alleged human cancer hazards based on irrelevant animal tests, yet those regulations remain enforced today. As noted, following the bioassay assimilation in WHO protocols, homologous regulatory practices expanded worldwide. If not enough, US federal regulatory programs metastasized into smaller competitive agencies with similar regulatory authority at state, cities and municipalities, vastly extending a mindset of autonomous administrative authority.

Clearly, the majority of people lack the tools and time to critically understand the current regulatory process, which has been endured as a matter of faith, likely for the same reasons the emperor is not seen as naked in the fable. In reality, a regulatory alliance appears to have anesthetized the collective conscience of nations into accepting whatever dictates are imposed under illegal claims of superior knowledge and authority.

Why illegal? Again, regulations in most democratic societies are equivalent to laws and laws can only be issued by freely elected legislative bodies. Legislators permit government agencies to impose regulations if based on evidence generally recognized as scientifically verified. Legislators grant this permission expecting to concur with such evidence and to act accordingly. By contrast, compulsory regulations would be illegal if issued autonomously by agencies on the basis of arbitrary judgments. Most recently, the US Supreme Court reaffirmed this doctrine, finding the USEPA acted illegally in setting arbitrary standards on greenhouse gas emissions from power plants: a weighty judgmental appraisal which the Court found to be the prerogative of legislators and not of unelected operatives.^15^

This verdict of the US Supreme Court ratifies the illegality of arbitrary justifications of massive regulatory burdens in the US, from default assumptions to safety factors and other imposed elements of risk assessment. Principal among these elements is the overused and supremely judgmental invocation of precaution, whose pervasive consequences are the central pretext for the self-serving illusory regulations of alleged human hazards on many fronts, from carcinogens to endocrine disruptors, pandemic management, global warming, and more.^16^ Precaution can be most expensive in terms of immediate and deferred opportunity costs. At the same time, definitions of its sensible extent are exclusively judgmental, especially when the targets of precaution cannot be factually assessed, as is the case with alleged hazards.

At large, this US Supreme Court ruling carries wide implications, for a cascade effect could upset much of the regulatory house of cards in the US and possibly worldwide. An earlier effect of this decision would be reminding legislators of their duty to critically assess whether regulations are justified by reliable evidence or by precautionary judgment, which could alarm legislators aware of their limited technical experience. A sensible solution would be requiring agencies to justify regulations by following legislator-approved evidentiary rules, thus relieving legislators from detailed technical hearings. In fact, such evidentiary rules are readily available in the Daubert decision of the US Supreme Court, which outlines the criteria of reliable evidence: the litmus test of legal and acceptable regulations.^17^ When trusted evidence under Daubert were not available, precautionary regulations may be considered on the basis of legislator-approved accounting criteria, to define the lesser exposure compatible with desirable uses under different exposure scenarios.

Notably, the Daubert ruling has been hailed as a turning point in liberating court proceedings from “*ipse dixit*” dogmatic experts who dominated judicial history, instead imposing the adoption of tested scientific evidence as the exclusive grounds of expert testimony. Daubert settled the first official confrontation of the rhetorical ways of a millennial legal tradition versus the testable evidentiary power of experimental science. Science won with Daubert, making for a crucial civilizing advance in human affairs.

## Moving ahead

Future commentators could not fail to marvel at the public quiescence facing today’s profoundly corrupt regulatory apparatus, hinged on alleged cancer hazards. Are we the same who send astronauts to the moon, develop wondrous electronics, conquer diseases, create the means to affordable food for billions, and much more? Change is overdue, for the just described regulatory arrangement is the ultimate breach of trust in government.

It would be presumptuous to chart a detailed course of remedial action, likely to happen by fits and starts, encouraged or repressed by evolving social and political models. Yet, it is possible to think of interventions toward testable and truthful evidence supporting fair regulations in health, safety, the environment and other scenarios as well.^18^

As noted, the US Administrative Practice Act list of agency misbehaviors would be sufficient to *“…set aside agency action, findings, and conclusions*” and should carry enough authority to dismiss the absurd construct now regulating alleged cancer hazards. Sensibly upgraded, the Act could have immediate effect by instructing regulatory agencies to exclusively adopt Daubert’s rules of evidence, integrated with the criteria of scientific evidence listed earlier in this paper, as the central effort toward a transparent and accountable government.

Such an approach would eliminate the illicit use of animal bioassays and the ensuing regulations, and force to reassess the current administrative setup presiding over the delusions of what is passed as regulatory science. As a desirable corollary, separate legislation protecting ethical transparency should also attempt to moderate or counter advocacy claims of alleged hazards apt to inflame unwarranted public anxieties. In the US, such claims and anxieties may prove inevitable in the superior context of constitutionally protected speech. Still, crying fire in a crowded theater without true evidence cannot be protected, which could justify requiring advocacies to sustain their claims under Daubert rules of evidence, or to underscore their hypothetical vagueness.

A general response for health, safety and environmental regulations would adopt separate guidelines for:

- Regulations backed by independent and testable scientific evidence, derived from acute and sub-acute tests in animals and humans, and from human epidemiologic studies counterfactually verified; and
- Precautionary regulations backed by utility considerations to permit only the least exposures necessary for effective uses, when hazards are only supposed and factual evidence is not achievable.

In this last context, a stark but obvious reality postulates the impossibility of life without hazards and risks, measurable or not, coupled with the obvious calamities of uncompromising precaution. It should be sensible to trade off the costs and uncertain safety of precaution versus the public health advantages of an enterprising commonwealth. At the same time, due to their exclusively judgmental makeup, precautionary regulations will likely require major input from elected legislators. As noted, statutory accounting guidelines could direct regulators in exploring precautionary opportunities, which would require new administrative staff training and expertise. A policy of precaution could be further assisted by a national epidemiologic surveillance program to monitor early signs of possible adverse effects in heavily exposed cohorts, similar to what is currently done in the pharmacovigilance of medicines and medical devices, and in occupational surveillance.

Additional remedies may include a Constitutional clarification of government authority and behavior in service to the public, a code of personal responsibility for government operatives of executive level, and expanding agency oversight by an independent judicial entity or an impossibly neutral executive agency. Moving in these directions would reaffirm the superior integrity of science and restore public trust in government, regulations and policies. The odds it may happen soon are low, for standing legal and bureaucratic constructs would put forth a mighty inertia in defending the indefensible status quo. Tied to current regulations, so numerous structural and personal economies are bound to resist changes to lifestyles, professional and institutional standings, records of past conduct, beliefs and pride.

Regulated industries—big names especially—also may resist, for compliance with an imposed regulatory ritual has continued to secure profitable marketing under however unwarranted government authority. Change could also upset the industrial reliance on the ritual’s high cost as an effective tool to moderate competition. Passionate resistance also could be expected from the Torts Bar, far too successful in riding the current arbitrary regulation of alleged hazards. Resistance could also arise worldwide, where illusory US practices were adopted uncritically and aggressively by WHO agencies and a majority of regulatory operators.

Yet, sooner or later, the current setup must change, since government impositions based on fabricated claims of objectivity cannot stand unchallenged in societies claiming and desirous to be enlightened and free. The vast human and material resources now wasted by regulations without verifiable social, public health and environmental benefits ought to be made productive, sustained by the discipline of science and the pledge of fairness, reason and citizen sovereignty, first set forth by “We the People of The United States” in their Constitution.

## Data Availability

This commentary article does not provide novel experimetal data, but simply draws conclusions based on diverse and previously published reports, the most significant being listed in the references.

## Footnotes

1 The Constitution of the United States of America. https://constitution.congress.gov/constitution/.

2 Human epidemiology equally has preventive interests in correlating cancer frequencies and exposure conditions in human cohorts. Not treated here are epidemiology’s similarly problematic methods and evidentiary approaches, which clearly differ from those of toxicological studies in animals, the limited interest of the present paper.

3 Substantial excerpts of this paper derive from *Aschner M. *et al*. Upholding science in health, safety and environmental risk assessments and regulations. Toxicology 2016;371:12–16*, which provided the technical foundations for the present article.

4 3. Deviations from this tenet would be judgmental and would require legislative assessment and approval of eventual precautionary regulations. More on this subject is addressed later in this article.

5 Daubert v. Merrell Dow Pharmaceuticals, Inc. (1993) 509 U.S. 579, 589. https://www.supremecourt.gov/opinions/boundvolumes/509bv.pdf.

6 The therapeutic promises of medicines and medical devices are ethically tested in human clinical trials to provide relevant reliable scientific evidence. However, medicines and medical devices are also tested in animals for possible long-term or chronic adverse effects.

7 – Gori GB. The Regulation of Carcinogenic Hazard. Science. 1980 208:256-261. – Gori GB. Science, Imaginable Risks, and Public Policy: Anatomy of a Mirage. Regulatory Toxicology and Pharmacology. 1996;23:3–4–311. – Gori GB. Regulatory Forum Opinion Piece: Long-term Animal Bioassays: Is the End Near? Toxicologic Pathology 2013;41:805-807. – Aschner M. et al. Upholding Science in Health, Safety and Environmental Risk Assessments and Regulations. Toxicology 2016;371:12–16 – An Appeal for the Integrity of Science and Public Policy. Toxicology 2016;371:12-16. – Cohen SM. The relevance of experimental carcinogenicity studies to human safety. Current Opinion in Toxicology. 217;3:6–11.

8 U.S. Environmental Protection Agency. Guidelines for Carcinogen Risk Assessment. EPA/630/P-03/001F. Risk Assessment Forum, U.S. Environmental Protection Agency, Washington, DC. March 2005. https://www.epa.gov/sites/default/files/2013-09/documents/cancer_guidelines_final_3-25-05.pdf.

9 Surprisingly, a precautionary mandate is enshrined in the regulatory practice of the European Union: https://www.europarl.europa.eu/RegData/etudes/IDAN/2015/573876/EPRS_IDA(2015)573876_EN.pdf.

10 National Research Council; Science and Decisions. Advancing Risk Assessment. National Academy Press. 2009. p.192 http://www.nap.edu/catalog.php?record_id=12209

11 The several years and $millions required to run a single cancer bioassay in rats and mice have been a constant frustration to official regulatory ambitions. Over the last decades, agencies have promoted less costly and rapid tests in cultured cells and organoids, to yield early signals of “adverse effects” of unknown significance to human health and disease. Those signals have entered regulation as minor corroborating corollaries to traditional schemes of hazard labeling, but clearly have the potential of opening the floodgates to destructive regulations if widely used in an alleged/precautionary context.

12 Administrative Procedure Act. Pub. L. 79–404, 60 Stat. 237, June 11, 1946. http://www.justice.gov/sites/default/files/jmd/legacy/2014/05/01/act-pl79-404.pdf.

13 Administrative Procedure Act. Section 7, (c) Evidence. http://www.justice.gov/sites/default/files/jmd/legacy/2014/05/01/act-pl79-404.pdf.

14 Administrative Procedure Act. Section 10, (e). Scope of Review. http://www.justice.gov/sites/default/files/jmd/legacy/2014/05/01/act-pl79-404.pdf.

15 West Virginia et al. v. Environmental Protection Agency et al.-Supreme Court of the United States. Decided June 30, 2022*. https://www.supremecourt.gov/opinions/21pdf/20-1530_n758.pdf.

16 Autrup H, et al.; Human exposure to synthetic endocrine disrupting chemicals (S-EDCs) is generally negligible as compared to natural compounds with higher or comparable endocrine activity: how to evaluate the risk of the S-EDCs? Arch Toxicol. 2020 Jul;94(7):2549-2557. https://doi.org/10.1007/s00204-020-02800-8 Koonin, Steven E.; Unsetted? What Climate Science tells us, what it doesn’t, and why it matters.; BenBella Books Inc., Dallas,TX; 2022.

17 Daubert v. Merrell Dow Pharmaceuticals, Inc. (1993) 509 U.S. 579, 589. https://www.supremecourt.gov/opinions/boundvolumes/509bv.pdf

18 Complex and diverse modes of action not only operate in causing cancer, but also for cardiovascular and neurological deficits, endocrine disruptions, aberrations of reproduction and immunity, and other anomalies triggered by random events over long timeframes. In humans under natural low exposure conditions, there are no methods to verify the alleged causal roles of most anomalies randomly associated with diseases of old age.

